# Mechanics of Brain Tissues Studied by Atomic Force Microscopy: A Perspective

**DOI:** 10.3389/fnins.2019.00600

**Published:** 2019-06-14

**Authors:** Prem Kumar Viji Babu, Manfred Radmacher

**Affiliations:** Institute of Biophysics, University of Bremen, Bremen, Germany

**Keywords:** tissue morphology, tissue mechanics, atomic force microscopy (AFM), tissue imaging, mechanical mapping

## Abstract

Tissue morphology and mechanics are crucial to the regulation of organ function. Investigating the exceptionally complex tissue of the brain at the sub-micron scale is challenging due to the complex structure and softness of this tissue, despite the large interest of biologists, medical engineers, biophysicists, and others in this topic. Atomic force microscopy (AFM) both as an imaging and as a mechanical tool provides an excellent opportunity to study soft biological samples such as live brain tissues. Here we review the principles of AFM, the performance of AFM in tissue imaging and mechanical mapping of cells and tissues, and finally opening the prospects and challenges of probing the biophysical properties of brain tissue using AFM.

## Introduction

Brain tissue combines an ensemble of different cells such as neurons and glia cells and the extracellular matrix, the latter is mainly made from filamentous proteins such as collagen, fibronectin, elastin, and others like proteoglycans and polysaccharides. Tissue mechanics results from the mechanical properties of the cells and the extracellular mechanics interacting with each other. So far, brain tissue mechanics has been investigated by various techniques such as atomic force microscopy (AFM) (Bouchonville et al., 2016), magnetic resonance elastography (MRE) (Mariappan et al., 2010), and ultrasound elastography (Gennisson et al., 2013). Among all, AFM has the advantage of allowing simultaneous imaging, mapping the mechanics with high resolution (nanometer scale precision), and force sensitivity (piconewton precision) of most tissues (brain, blood vessel, lung, cartilage, tendon) in either fluids or physiologically relevant environments (Mao et al., 2009; Chan et al., 2010; Liu and Tschumperlin, 2011; Marturano et al., 2013; Iwashita et al., 2014). The advent of AFM to capture the live actions of biomolecules at high spatial and temporal resolutions has been enabled by techniques such as high-speed AFM (Ando, 2018; Heath and Scheuring, 2019). AFM-based recognition imaging and force spectroscopy enables unbinding force mapping of receptors–ligand interaction sites on a lipid membrane at the single molecule level (Koehler et al., 2019). Not only a surface-imaging tool, but also a force–distance (FD) curve-based AFM has been used in different modes such as ringing (Dokukin and Sokolov, 2017), tapping (Zhong et al., 1993), multifrequency (Garcia and Herruzo, 2012), and contact resonance (Stan et al., 2014) mode to measure nanoscale mechanical (viscoelastic) properties of cells, biopolymers, and tissues. At the cellular level, single-cell force spectroscopy (SCFS)-based AFM adds extra information and is increasingly used to study cell mechanics (Lekka et al., 1999; Rianna and Radmacher, 2017; Viji Babu et al., 2018), cell–cell interaction (Benoit et al., 2000), and cell–ECM interaction (Friedrichs et al., 2010). Similarly, AFM has been used as an imaging and spectroscopic [single-molecule force spectroscopy (SMFS)] tool in investigating bio-molecular structures (Shibata et al., 2017) and their intra- and inter-molecular interactions (Florin et al., 1994; Neuman and Nagy, 2008). Not only restricted to their ability to measure forces and displacements accurately and precisely, AFM cantilevers which act as a spring were also used as a motion micro-sensor to detect nanoscale vibrations of various prokaryotic and eukaryotic cells (Kasas et al., 2015). From single-molecule to single-cell manipulation, AFM becomes a multifunctional toolbox to observe and measure various biophysical parameters of cellular and subcellular assemblies and machineries. Remarkably, AFM can be used in cell or biomolecule physiological conditions and also does not require elaborated or specific sample preparation. AFM provides a technology that can also be integrated with other microscopic and spectroscopic techniques such as laser scanning confocal (Staunton et al., 2016), Total Internal Reflection Fluorescence (TIRF) (Ramachandran et al., 2014), STimulated Emission Depletion (STED) (Harke et al., 2012), and Förster Resonance Energy Transfer (FRET) (He et al., 2012). These correlative approaches offer a wide spatial (nm) and high temporal (ms) resolution to study cellular and molecular biophysics. Currently, AFM has gained a lot of attention in the field of biomedical engineering, especially in investigating the mechanical properties of tissues. Researchers take advantage of the simple sample preparation in AFM, which allows studying the living samples surface through imaging and mechanical mapping at the same time. In cancerology, AFM has been extensively used as an innovative diagnostic tool to explore the effects of cytotoxic drugs (Pillet et al., 2014). With simple setup and principle, AFM probes the tissue dynamics at the nano-scale.

The presence of different types of cells and their correlated functions including ECM synthesis, remodeling, and degradation (mainly fibroblasts) makes a tissue (connective tissue) unique within an organ. So far, biochemical properties of tissues have provided a large amount of information about the presence of tissue or cell specific biomarkers. These biomarkers reveal the distinction between the healthy and diseased state of a tissue, which may help in synthesizing specifically targeted drugs. Cell mechanics has now become a potential biomarker to discriminate between the different physiological and pathological states of cells (Rianna and Radmacher, 2016). Similarly, investigating tissue mechanics opens up a new platform in the biomedical field to diagnose pathological states of different tissues.

Generally speaking, brain tissue has three distinct parts: the cerebrum, cerebellum, and the brainstem. Each part has its own unique function in governing the different functions of the human body. As the central nervous system (CNS) for the whole body, brain tissues mainly contain neuronal and glia cells which interact through electric and ionic signaling and neurotransmitters. The mechanical properties of neurons and glia cells play a key role in neuronal growth and development (Spedden and Staii, 2013). Studying local and global brain topography and mechanics noninvasively can lead to a better understanding of the development of various diseases such as neurodegenerative diseases and cancer. Previous rheological studies on brain tissues were mostly conducted non-destructively on a macroscopic scale of centimeter to millimeter. Investigation into micro- and nano-scale range regions of living brain samples may allow distinguishing between cell and ECM properties and their correlation.

The main goal of this mini review is to introduce readers to the working principle of AFM and its application in tissue imaging and the mapping of mechanical properties of tissues. Finally, we discuss the possibility of using AFM in brain tissue biomechanics.

## AFM – Working Principle – Imaging and Mechanical Mapping

**FIGURE 1:**
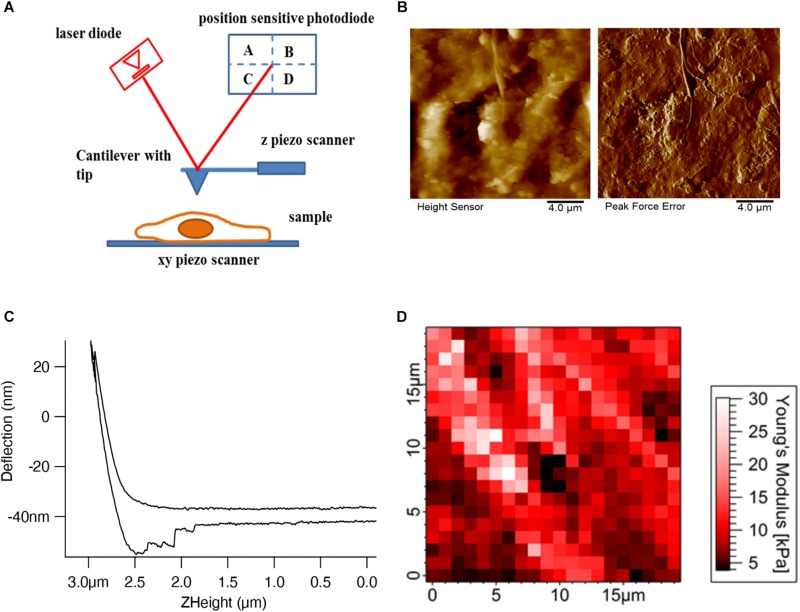
AFM setup and tissue imaging. **(A)** AFM consists of four important components – cantilever with an AFM tip, laser diode, position-sensitive photodetector, and xyz-piezo scanner. **(B)** Height and deflection images show the decellularized dermal matrix which shows the collagen fibers running along other matrix components (Rianna et al., 2018). **(C)** Deflection (nm) vs. Z height (μm) shows the approach and retract curve (Viji Babu et al., 2018). **(D)** Force maps show the elastic modulus values of mouse skin tissue in a respective color scale (Pa) (Joshi et al., 2017).

Atomic force microscopy is conceptually a simple technique, employing the interaction between a tip whose shape can be tuned according to the application (sharp tips for high resolution imaging and pyramidal or spherical tips for mechanical mapping) attached to a soft cantilever spring and the sample. There are four main components (Figure 1A) in AFM: a cantilever, which acts as a spring with an integrated tip; a laser beam focused onto the very end of the cantilever where the tip is attached; a position-sensitive photo-detector to detect the reflected laser beam, which can measure the horizontal and vertical deflection of the cantilever; and finally, a xyz piezo scanner for moving the sample or the cantilever in all three directions. In our example schematics, the piezo scanner setup has been designed in such a way that the z piezo controls the cantilever movement in the z-direction and the xy piezo controls the sample movement in the xy-direction.

Different imaging modes such as contact (DC) and non-contact tapping (AC) modes are used in AFM to measure the sample topography. In contact mode, the AFM tip is brought into physical contact with the sample and the cantilever deflection is measured. In the constant height mode, the sample is kept at a constant height while the tip raster scans the sample. The topographic information is inferred from the deflection of the cantilever as the tip scans over areas of different heights. This particular mode is generally used for flat and rigid samples, since, due to the deflection of the cantilever the loading force will change. For soft biological samples, especially for cells, this mode will damage the cells as they will be exposed to large loading forces. In order to image soft samples, a feedback is introduced to adjust the z height such that the deflection, and therefore the loading force, is held constant. This mode is called a constant force or constant deflection mode. Figure 1B shows the height and error signal images of the extracellular matrix topography of the decellularized dermal matrix. In constant deflection mode, the output of the feedback corresponds to the height signal image which shows the overall sample topography. Since the feedback will react with a finite response time, the main time limiting factor will be the piezo transducers used in AFM, there are some residual changes in deflection, which are not perfectly compensated. In control theory this behavior is called the error (of the feedback loop); therefore, in AFM the phrase error signal image is also often used. To reduce lateral forces exerted to the sample in contact mode, which can be substantial and destroy or detach samples, the tip is periodically retracted from the sample and the cantilever height is modulated at the cantilever’s resonance frequency. This mode is called the tapping mode and is used largely in imaging biomolecules such as DNA, proteins, and lipids. Like in contact deflection mode, tapping mode produces two images: a height and an amplitude error image. A novel variant of the tapping mode, the peak force mode, where the data during one oscillation cycle are captured and analyzed online to control the maximum force, seems to be favorable for cell imaging (Figure 1B; Schillers et al., 2016).

In a force curve (Figure 1C) the interaction forces between the tip and sample are measured while the tip is approached and retracted from the sample. This can be performed over a region of interest of the sample, generating a force map or force volume (Figure 1D) in which each pixel in the map represents a force curve. Both the approach and retract curves reflect information on the mechanical, or more precise viscoelastic properties of the sample, as well as adhesion properties between the tip and sample, e.g., a cell or the ECM. The elastic properties of the sample can be inferred by fitting the data with an appropriate geometric model of the tip and sample to yield the Young’s modulus. Different models are used from continuum mechanics depending on the shape of the AFM tip. In most cases, the AFM tip shapes are pyramidal, conical, and spherical. According to the tip geometry, the Hertz model (Hertz, 1882) for spherical indenters, Sneddon model (Sneddon, 1965) for conical indenters, and Sneddon extended model (Rico et al., 2005) for pyramidal indenters are used to describe the elastic behavior of the biological samples. The force can be calculated by Hooke’s law from the deflection of the cantilever, if the spring constant is known. The sample indentation is calculated from the z movement of the z piezo and the cantilever deflection.

## Tissue Imaging and Mechanical Mapping in AFM

The simplicity of the working principle of AFM allows users to obtain the fine microstructures of biological tissue with good resolution. Biological tissues are comprised of different cells and ECM, whose interplay facilitates tissue dynamics and maintains homeostasis. Investigating biomechanical properties and imaging of cells and ECM are studied individually and cells are mostly cultured in hydrogels, matrigels, or three-dimensional (3D) matrices in order to evaluate the substrate stiffness or composition-dependent cell elastic properties. Whereas decellularized ECM is evaluated for the ECM component arrangement and stiffness. Intracellular actin cytoskeleton arrangement and dynamics reveal that the cell stiffness and actin stress fibers interact with and transmit mechanical information to the ECM through the transmembrane protein focal adhesion complex. This adhesion complex consists of the transmembrane protein integrin, whose extracellular domain binds to the RGD (Arg-Gly-Asp) sequence of any of the ECM proteins and its intracellular domain binds to the adaptor proteins which further bind to the actin cytoskeleton. This combined complex transfers both the extracellular and intracellular force generated by respective ECM protein fibers and actin stress fibers in the cells, resulting in signaling in both directions: from the cell to the ECM environment and back from the environment into the cells (Discher et al., 2005). ECM imaging and biomechanical properties are so far performed in decellularized tissue samples. The surface topology of acellular ECM scaffolds provides information on the ECM protein fibers’ orientation, spacing, diameter, and also records mechanical maps which enable their stiffness and surface roughness. Collagen fibers are mostly dominant and abundant in these decellularized tissue samples and sometimes the collagen fibers are also seen with other ECM proteins. AFM measurement can be combined with fluorescence microscopy in order to study different ECM proteins by tagging them with different fluorophores which make them easier to visualize in conducting decellularized ECM imaging and mechanics (Jorba et al., 2017).

**FIGURE 2:**
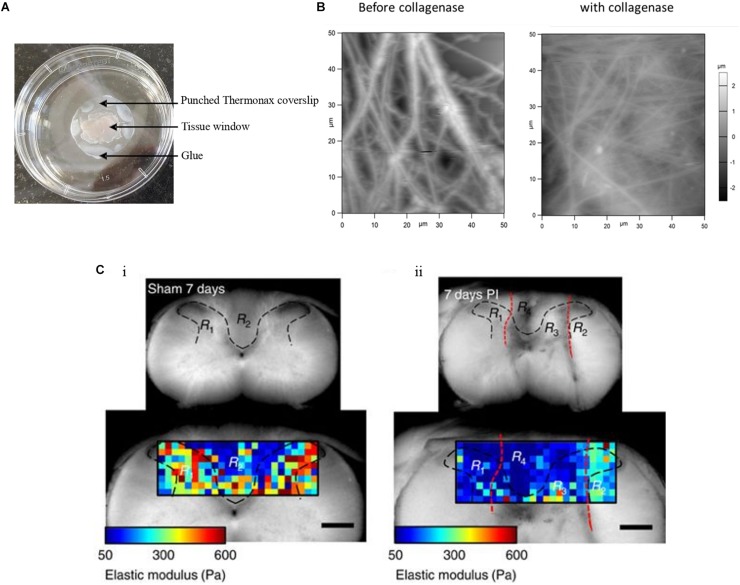
Tissue sample immobilization and AFM imaging of mouse skin tissue sample. **(A)** Tissue samples are immobilized with punched Thermanox coverslips, glued to the Petri dish at their borders, thus avoiding direct contact of tissue with glue. AFM tip accesses the sample through the tissue window. **(B)** AFM height images show the presence of thick and rich ECM fibers in the mouse skin tissue matrix before addition of collagenase and after the addition of thick fibers, after which disappeared and decreased ECM fibers are seen (Joshi et al., 2017). **(C)** The gray (R_1_ and R_3_ in both i and ii) and white (R_2_ and R_4_ in both i and ii) matter are indicated within the area (black dashed lines) of the transverse spinal cord section of a sham control and of an animal with a dorsal column crush lesion at 7 days post-injury. The elastic moduli values are represented by means of a color map. In ii, the injured areas are identified by the red dashed lines (Reprinted with the permission from Moeendarbary et al., 2017 and this work is licensed under a Creative Commons Attribution 4.0 International License. To view the copy of this license, visit http://creativecommons.org/licenses/by/4.0/).

Ensemble investigations of cells and ECM at the tissue or sub-tissue level provide information on the cell–ECM mechanical crosstalk and disease-related alterations in tissue morphology and mechanics. The AFM sample preparation for tissue investigation starts with the immobilization of tissue blocks which is quite challenging. Tissues are normally immobilized to a coverslip or any other suitable support in several ways. Tissue adhesives such as Histoacryl tissue glue (Stolz et al., 2009; Plodinec et al., 2010) or ethyl cyanoacrylate (Chan et al., 2010) are mostly used and care has been taken so that adhesives do not make contact with the investigated region. Nevertheless, this immobilization procedure will have some effect on the tissue as substances released from the adhesives may diffuse, either through the surrounding air or water, or directly through the neighboring tissue. Sectioned tissue specimens are often immobilized to microscope glass slides coated with poly-lysine (Stolz et al., 2004; Grant et al., 2012). This procedure is a good option but only for thin tissue sections. The main aim of immobilization of tissues is to avoid sample movement while recording images or mechanical maps. An alternative to adhesives is using Thermanox coverslips punctured in the center and used for holding down the sample in such a way that the tissue can be accessed by the AFM tip. The edge of the Thermanox coverslip can then be glued to the support (Figure 2A; Morgan et al., 2014). This setup avoids contact of tissue and glue or any other adhesive materials and serves as a better way to immobilize tissue samples. This approach paves way for investigating the topographical and mechanical changes of the mouse skin tissue. Figure 2B shows the presence of thick ECM fibers in mouse skin tissue before and after the addition of collagenase, which leads to the disappearance of fibers and correlatively decreases their elastic properties (Joshi et al., 2017). Biological tissues, cells and the ECM, are composed mainly of water (around 70% for the cytosol), even though for the biological function usually only macromolecules such as proteins or small organic and inorganic molecules are discussed. Therefore, it is very important to study tissues in a hydrated environment such as selecting the suitable medium which brings the utmost native environment to tissues that affect their topography and mechanical properties. It was reported (Zhu and Fang, 2012) that hydration and dehydration of cartilage affects the collagen fiber distribution and its roughness. Standardizing the tissue immobilization protocol and selecting the correct liquid medium are simple to set up and efficient in measuring the morphological and mechanical alteration in the tissues. Tissue samples are normally very soft. As a consequence, they are very difficult to cut into thin slices in their native state even with state of the art vibratomes; therefore, they are often frozen to prepare thin sections (cryosectioning). Normally thin tissue sections of 5–50 μm in thickness are generated for imaging and recording mechanical maps in AFM studies. However, the cryo-procedure not only decreases the cell viability dramatically, but also changes ECM and cell mechanics. Thus, the interplay of cell and ECM mechanics will be difficult or even impossible to investigate. A recent report (Xu et al., 2016) demonstrated the ability to show the difference in mechanical properties of vibratome and cryotome tissue sections. Vibratomed tissue sections show good cell viability and in mechanical maps, cell and ECM regions can be distinguished. Thus, in vibratomed sections nearly all the properties of the living tissue sample are preserved, e.g., for AFM measurements. In contrast, in mechanical maps recorded from cryotomed sections, cell and ECM regions could not be distinguished, because the freezing process increased the stiffness of the entire tissue, possibly because cells were not viable anymore.

For AFM imaging, cryotomed tissue sections or samples chemically fixed in paraformaldehyde are mostly used. Both preparations increase sample stiffness and decrease adhesion to the cantilever tip (Joshi et al., 2017), which makes them suitable only for imaging; mechanical data from chemically fixed samples will be strongly affected by this sample preparation and show no resemblance with the mechanical data of live cells or tissue (Braet et al., 1998). Tissue sample preparation, immobilization, and hydration procedures are important for imaging and for obtaining mechanical maps of native samples by AFM. In a pioneering work it has been demonstrated that AFM can be used for *in vivo* nanomechanical imaging in living mammals by capturing real-time changes in nanomechanical properties during vasodilation and vasoconstriction in blood vessels (Mao et al., 2009). Although it is not clear how this could be adopted to other applications, especially in humans, it clearly demonstrates the potential of AFM directly following dynamics in living mammalian tissues. AFM was also used for capturing ultrastructural high-resolution imaging of native biomolecules, specifically intracellular organelles and extracellular matrix structures in mammalian connective tissue cryo-sections (Graham et al., 2010). AFM nanoindentation experiments are able to characterize the stiffness and elastic modulus of soft tissue scaffolds such as collagen–chitosan biopolymers (Zhu et al., 2011). 3D mechanical properties of the skin epidermis and dermis at the nanoscale resolution are measured using AFM nanoindentation (Kao et al., 2016). By selecting the right AFM probes and by standardizing tissue section preparation and immobilization, one can measure the morphological and biomechanical changes in the tissue of any animal.

## AFM in Neurobiology – Prospects and Challenges

Here we discuss some of the reports where AFM was used in neurobiology, concentrating on neuronal and glial cells’ biomechanics, including also brain tissue mechanics. For a detailed review on AFM usage in neuron biomechanics, readers are pointed to this review (Spedden and Staii, 2013). In neuronal growth and development, both neuronal cells and ECM function in a very coordinated manner. For example, the intracellular actin cytoskeleton helps neuronal growth cone movements. During development, neuronal growth cones are found at the apex of each axon and move in a direct way toward the target cell through the detection of extracellular signals. High resolution imaging and by studying the mechanical properties of these neuronal growth cones using AFM show varying protein motilities between different growth cone regions (Grzywa et al., 2006). AFM imaging of neurons grown on laminin micropatterns shows the laminin-guided neurite growth and the role of actin cytoskeleton in growth cone dynamics (Xing et al., 2010). AFM combined with confocal fluorescence microscopy have been used to analyze the morphology of neuronal growth cones of rat dorsal root ganglia (Laishram et al., 2009). 3D imaging from AFM and confocal evaluates the 3D architecture of living chick dorsal root ganglia and sympathetic ganglia (McNally et al., 2005). 3D AFM imaging of neurons and glia cells provides a wealth of information on 3D cell structures and sub-cellular structures of organelles such as the mitochondria or the nucleus (Parpura et al., 1993). Viscoelastic properties of individual neuronal and glial cells in the CNS show a large mechanical heterogeneity because of the distribution of cell organelles. Generally, both cell types are very soft compared to other eukaryotic cells (Lu et al., 2006). This work also reveals that glial cells do not serve as a structural support or as a glue cell for neuronal cells. In all the reports mentioned above, chemically fixed or living cells are used for AFM imaging and mechanical measurements.

The discussion of AFM application in brain cells opens up the possibility of mechanical characterization of brain tissues using AFM. Before we further discuss this topic, the focus on tissue sample preparation for such application has to be elaborated on as this provides varied techniques along with their advantages and disadvantages. Tissue extraction, embedding, and slice preparations largely fall into deformations, due to the loss of the native environment and dehydration. This causes global shrinkage from the earlier primary and secondary deformations and greatly affects the tissue structures (Dauguet et al., 2007). This demands a unique embedding and slice preparation method to maintain the brain tissue integrity. Concerning 2D brain tissue preparation, conventional slice preparation methods for AFM investigation largely alter the tissue structure. Therefore, agar embedded tissue blocks were used for slide preparation which maintains the tissue structure and helps in carrying out mechanical measurements (Iwashita et al., 2014). Regarding 2D tissue section mechanical preservation, AFM mechanical characterization of living tissues showed cryotomed sections preventing the mechanical measurements of cells in the tissues. Comparatively, vibratomed sections are able to distinguish between the cell and ECM regions in the AFM-generated sub-micron resolution mechanical maps (Xu et al., 2016). As brain tissues are much softer than other tissues, their sectioning preparation by vibratome in a standardized fashion is quite challenging. Earlier, our lab tried to prepare cancer tissue sections from vibratome and due to the softness of the cancer tissue, we could not succeed in obtaining reproducible and useful results. The preparation of brain tissues from techniques other than cryotome may be advantageous to measure living tissue’s mechanical properties.

Sub-tissue level nanomechanical imaging of both cells and ECM could possibly demonstrate the elastic properties as well as fine details of biomolecular structures of different brain regions. AFM measurements of the hippocampal and cortex regions of a rat brain show mechanical heterogeneity in subregions and also age-dependent tissue stiffness correlation (Elkin et al., 2007; Elkin et al., 2010). AFM indentation, with a spherical indenter, shows significant mechanical differences between white and gray matter of the rat cerebellum (Christ et al., 2010). AFM spatiotemporal tissue mechanical profiling shows the gradual stiffness increase in ventricular and subventricular zones of a mouse brain during embryonic development (Iwashita et al., 2014). Spatial mechanical mapping of the living guinea pigs’ retinae, using scanning force microscopy, determines the elastic modulus of retinal regions and finds the contribution of neuronal cell bodies to the mechanical properties of inner retina (Franze et al., 2011). At the microscale, AFM time lapse *in vivo* imaging was performed in a live *Xenopus laevis* embryo in order to follow their local stiffness, which changes during embryonic brain development. This change in brain local stiffness is largely due to brain cell proliferation (Thompson et al., 2019). This time-resolved stiffness measurement can be conducted in other tissue development and is able to capture the tissue dynamics in varying temporal resolutions. Other than following embryonic brain development, AFM mechanical maps show the mechanical heterogeneity in a mouse primary somatosensory cortex and their age-dependent increase in tissue stiffness. Furthermore, AFM topographical imaging of thin sections of the different layers of the sensory cortex at different ages (weaning to adulthood) shows the continuing smoothing of the cortex surfaces (Smolyakov et al., 2018). AFM is a useful tool that can elucidate stiffness maps which correlate to brain development and many neurodegenerative diseases. AFM mechanical measurement shows Alzheimer’s disease associated reduced brain tissue stiffness in mice comparatively to their wild type in both normoxia and hypoxia conditions (Menal et al., 2018). The molecular mechanism of amyloid beta (Aβ) fibril formation and toxicity in Alzheimer’s disease is well characterized by AFM imaging (Moores et al., 2011). Pathological conditions such as acidosis stiffens the cerebellar gray matter when brain tissue is exposed to CO_2_ thus decreasing the pH (Holtzmann et al., 2016). Comparative to other mammalian tissues, CNS tissue softens after injury. The glial intermediate filaments and ECM composition such as laminin and collagen IV contribute to the rat brain neocortex tissue softening assessed by AFM micro-indentation experiments (Moeendarbary et al., 2017). The elastic modulus color maps from gray and white matter regions show a decrease in elastic modulus values in the 7 days post-injured area, as compared to the control (Figure 2C). AFM mechanical characterization of a rat brain subjected to thromboembolic focal vertebral ischemia shows a decreased Young’s modulus compared to the wild type (Michalski et al., 2015). Lewy bodies from postmortem brain tissue samples of Parkinson’s disease human patients were imaged by AFM. These images show the aggregated fibrillary nanostructures in Lewy bodies and also show disconnected neurons which are located in the substantia nigra (Tercjak et al., 2014). The mechanical fingerprint of human glioblastoma and meningothelial meningioma tissues was measured by AFM mechanical maps. These potential applications of AFM in brain development and diseased tissue characterization help to better understand the sub-tissue level mechanosensitivity and its implications in the cells and ECM mechanical interactions. The recent advancement in AFM makes it an ideal tool to understand the role of mechanical cues in brain tissues and to correlate these cues to histopathological features. Further advancement in brain tissue sample preparation methods, together with AFM bioimaging techniques, widely covers the neuroscientific network by using AFM for diagnosing and analyzing neurodegenerative diseases.

## Conclusion

We reviewed the application of AFM bio-imaging and mechanical mapping of soft tissue samples like brain tissue and discussed the ability of AFM to work under near physiological conditions, which is essential for mechanical mapping. With the simple working principle, scientists from different disciplines can solve arising questions in tissue biology with the aid of AFM. The challenges in tissue sample immobilization, using the right liquid medium and tissue sectioning for AFM experiments were also discussed. As outlined and reviewed, medical engineers and scientists have to keep the challenges mentioned above in mind, and design experiments accordingly to study different tissues of varying animals. Concerning the brain tissue, there is great demand to use AFM in brain tissue imaging to visualize the micro scale arrangements of cells together with ECM. Additionally, obtaining mechanical maps of different regions of the brain enables one to study varying stiffness within brain tissues.

## Author Contributions

PV performed the AFM experiments, data analysis, and manuscript preparation. MR designed the content and was involved in data analysis and preparation of the manuscript.

## Conflict of Interest Statement

The authors declare that the research was conducted in the absence of any commercial or financial relationships that could be construed as a potential conflict of interest.
